# Epidemiological shifts and advances in research on early diagnosis of invasive fungal infection in critically ill patients

**DOI:** 10.3389/fcimb.2025.1658476

**Published:** 2025-12-11

**Authors:** Yuan Cao, Yun Li, Min Wang, Yiqi Wu, Shaoqing Shi, Chengjin Wang, Zhen Gao, Wenjun Yang, Lu Wang, Hongjun Kang

**Affiliations:** 1 Medical School of Chinese PLA, Beijing, China; 2 Department of Critical Care Medicine, the First Medical Centre, Chinese PLA General Hospital, Beijing, China; 3 General Hospital of Southern Theater Command of PLA, Guangzhou,, China; 4 Department of Critical Care Medicine, Fifth Medical Center of Chinese PLA General Hospital, Beijing, China

**Keywords:** *Aspergillosis*, candidiasis, fungal detection, intensive care unit, invasive fungal infection

## Abstract

Invasive fungal infections (IFI) primarily occur in immunocompromised patients, particularly those in intensive care units (ICU). Due to the use of immunosuppressive agents, invasive therapeutic procedures, and advancements in diagnostic technologies, the detection rate of IFI has shown a significant upward trend. This review aims to explore epidemiological changes in the field of IFI, early detection techniques, and the application of artificial intelligence (AI) technologies. We conducted a literature review using PubMed data up to April 2025, focusing on studies related to IFI. Specifically, we focus on three aspects of IFI research: first, the epidemiology of IFI is undergoing significant changes, with *Candida auris* rapidly spreading across more than 40 countries worldwide, and rare fungal infections such as *Mucor* spp. and *Fusarium* spp. becoming increasingly prevalent; simultaneously, resistance to antifungal drugs among various pathogens continues to rise. Second, breakthroughs have been achieved in early detection technologies, including molecular detection techniques, biomarker testing, imaging technologies, and other emerging diagnostic methods, significantly enhancing the sensitivity and specificity of diagnosis. Thirdly, with the widespread application of AI technology, the development of clinical predictive models, the establishment of scoring rules, and the formulation of AI-based treatment decision-making tools are advancing the exploration of early diagnosis for IFI. In summary, as early diagnostic technologies for IFI continue to advance and AI algorithms are integrated into clinical practice, there is potential to improve the early diagnosis and treatment outcomes for critically ill patients with IFI.

## Graphical Abstract

**Figure:**
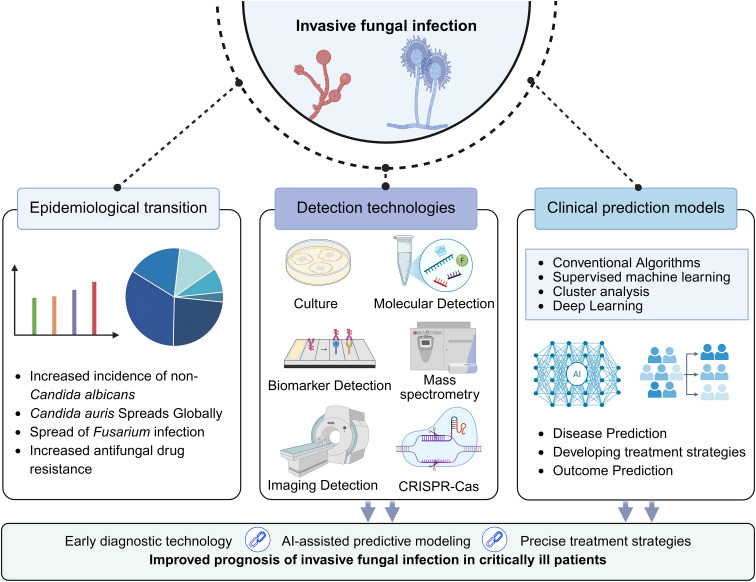
Epidemiological changes and early diagnosis of invasive fungal infections in critically ill patients.

## 1 Background

Over the past decade, the global burden of fungal infections has escalated significantly (Bongomin et al., 2017). With the widespread application of cancer chemotherapy, organ transplantation, immunosuppressive agents, and broad-spectrum antimicrobials, coupled with continuous advancements in detection technologies, the overall detection rate of Invasive Fungal Infection (IFI) demonstrates a persistent upward trajectory (Bretagne et al., 2022; Chen et al., 2018). The incidence of IFI across major healthcare networks in the United States increased from 30.2 per 100,000 individuals in 2006 to 31.8 per 100,000 in 2015 (Webb et al., 2018). In France, the rate of IFI rose from 2.16 to 2.36 per 10,000 hospital-days between 2012 and 2018 (Bretagne et al., 2022). In China, the overall incidence of IFI has steadily escalated in both immunocompromised and immunocompetent populations, accompanied by a notable rise in infections caused by uncommon fungal pathogens (Chen et al., 2018). However, due to the non-specific clinical manifestations of IFI and limitations in detection technologies, a substantial proportion of IFI remain undiagnosed and untreated in a timely manner, consequently establishing IFI as one of the frequently overlooked causes of mortality in Intensive Care Unit (ICU) (Clancy and Nguyen, 2013; Dignani, 2014). Although the causative fungal species vary slightly across geographical regions, *Candida* and *Aspergillus* species predominate, accounting for approximately 90% of all IFI. Risk factors for IFI development in ICU patients differ according to the infecting species. For invasive candidiasis (IC), the most common predisposing factors include *Candida* colonisation, mechanical ventilation, central venous catheterisation, parenteral nutrition, dialysis, pancreatitis, antibiotic therapy, and corticosteroid administration (Patterson et al., 2019; Thomas-Rüddel et al., 2022). Prominent risk factors for invasive aspergillosis (IA) encompass chronic obstructive pulmonary disease (COPD), prolonged corticosteroid therapy, hepatic cirrhosis, malignancy, HIV infection, and lung transplantation (Levesque et al., 2019; Gago et al., 2019). Epidemiological data indicate approximately 6.5 million cases of invasive fungal infections annually, resulting in over 3.8 million deaths, primarily attributed to *Aspergillus*, *Candida*, *Cryptococcus*, and *Pneumocystis* species (Denning, 2024; Thambugala et al., 2024). Mortality varies substantially depending on patient demographics, immune status, and the implicated fungal taxa. During the past decade, the crude 30-day mortality rate for IC in ICU has been approximately 40-55%, with candidaemia mortality rates reaching 71% (Bassetti et al., 2019; Xiao et al., 2019). Invasive mould infections demonstrate mortality rates of approximately 46-85% (Schauwvlieghe et al., 2018; Bretagne et al., 2022). In lung cancer, invasive aspergillosis accounts for approximately 40% of deaths; in COPD, mortality may reach 80%. Among patients with leukemia or lymphoma, as well as those undergoing allogeneic hematopoietic stem-cell transplantation, the mortality rate of invasive aspergillosis approaches 80% (Denning, 2024).

Despite increased global awareness of IFI, detection rates and therapeutic outcomes remain suboptimal. IFI frequently afflict immunocompromised populations, particularly ICU patients, where delayed diagnosis often portends adverse outcomes and increases hospitalisation burden. Early diagnosis facilitates prompt intervention and prevents further fungal dissemination. In this review, we endeavour to summarise the evolving epidemiological trends of IFI in critically ill patients, early detection technologies, and the development of clinical prediction and early warning models based on electronic health records and artificial intelligence (AI) algorithms, subsequently analysing future research directions in this field. The core content of this article is illustrated in Figure abstract.

## 2 Transformation of epidemiological trends

### 2.1 Increasing incidence of rare fungal infections

#### 2.1.1 Candida

*Candida* species represent the most prevalent fungal pathogens worldwide. Historically, *Candida albicans* has dominated invasive fungal infections. However, a comprehensive analysis conducted by Soulountsi et al. of studies spanning 2001 to 2020 revealed a decline in the incidence of C. albicans, accompanied by a persistent rise in non-*albicans Candida* (NAC) infections. Among these, *Candida glabrata, Candida parapsilosis*, and *Candida tropicalis* are the most frequently isolated species (Soulountsi et al., 2021). Large multicentre investigations and meta-analyses from Europe, North America, and the Asia–Pacific region indicate that NAC organisms currently account for approximately 40–60% of all candidemia cases (Arendrup et al., 2023). A longitudinal surveillance study in Italy (2015–2022) further demonstrated that beginning in 2019, *C. parapsilosis sensu stricto* surpassed *C. albicans* as the leading causative agent of bloodstream candidiasis, paralleled by a dramatic escalation in azole non-susceptibility—exceeding 50% by 2022 (Franconi et al., 2023). Epidemiological studies conducted in China similarly demonstrate that, although *Candida albicans* remains the predominant pathogen, the incidence of non-*albicans Candida* continues to rise, now accounting for more than 50% of clinical isolates. Notably, the prevalence of several previously rare species has surged over the past five years, including multidrug-resistant *C. glabrata* and *C. auris* (Soulountsi et al., 2021; Yu et al., 2024). These findings confirm that NAC species have assumed dominance across multiple geographic settings, reinforcing the imperative that antifungal therapeutic strategies be informed by regional epidemiological patterns. Furthermore, ongoing revisions in fungal taxonomy have resulted in the reclassification of several species formerly assigned to the *Candida genus*. *Candida lusitaniae* is now designated *Clavispora lusitaniae*, *Candida guilliermondii* as *Meyerozyma guilliermondii*, and *Candida famata* as *Debaryomyces hansenii* (Kidd et al., 2023). These nomenclature changes reflect advances in DNA-based phylogenetics and have implications for species identification in clinical laboratories, particularly when utilising molecular diagnostic platforms that may retain legacy nomenclature in their databases.

*C. auris* is an emerging multidrug-resistant fungal pathogen predominantly affecting immunocompromised patients, particularly those in ICU, functioning as an opportunistic pathogen. *C. auris* was first identified in 2009 from the external auditory canal of a female patient in Japan (Satoh et al., 2009). Between April 2015 and November 2016, an outbreak involving 72 patients occurred at a hospital in the United Kingdom (Rhodes et al., 2018). China first isolated a *C. auris* strain (BJCA001) in 2018, and since then, *C. auris* has been reported in numerous hospitals nationwide (Wang et al., 2018). The Centers for Disease Control and Prevention (CDC) tracked 63 clinical cases of *C. auris* infection between 2013 and 2016; by December 31, 2022, the total reported cases had escalated to 5,654 (CDC, 2024). Currently, *C. auris* infections span six continents and more than 40 countries and regions (Hu et al., 2021). *C. auris* can colonise diverse anatomical sites including the nasal passages, axillae, groin, and skin surface. Prolonged hospitalisation and previous exposure to antibiotics or antifungal therapy increase susceptibility to infection, with transmission occurring through contact or faecal spread. Once established, colonisation may persist for three months or longer (Jeffery-Smith et al., 2017). *C. auris* exhibits multiple virulence characteristics, including biofilm formation, production of adhesins and proteases, and the capacity to evade innate immune responses (Horton et al., 2023). Phenotypic microbial observation alone may inadequately differentiate *C. auris* from other *Candida* species; novel detection methods such as MALDI-TOF mass spectrometry enable more reliable and rapid identification (Spivak and Hanson, 2018). Due to its non-specific clinical manifestations and high antimicrobial resistance profile, *C. auris* infections demonstrate mortality rates exceeding 50%, prompting significant international concern within the infectious disease community (Lockhart et al., 2017).

Beyond *Candida auris*, several non-*albicans Candida* species exhibit distinct clinical and epidemiological characteristics. *Candida glabrata*, frequently ranking second in prevalence in many regions, has shown an alarming escalation in azole resistance, with fluconazole resistance rates reaching 13% in the United States (Pfaller et al., 2019; Lyman et al., 2023). *Candida parapsilosis*, predominantly transmitted via healthcare workers’ hands and contaminated medical devices, represents a leading cause of catheter-associated bloodstream infections, particularly in neonatal intensive care units and among patients receiving parenteral nutrition (Garzillo et al., 2017; Asogan et al., 2024). Owing to a natural proline-to-alanine polymorphism (P660A) within the hotspot region of the FKS1 gene, this species exhibits intrinsic reduced susceptibility to echinocandins (Garcia-Effron et al., 2008), further complicating first-line antifungal therapy. Recent global surveillance indicates that *C. parapsilosis* accounts for 15–30% of candidemia cases, with disproportionately high prevalence in Southern Europe and Latin America, and a global fluconazole resistance rate of 15.2% (Lamoth et al., 2018). *Candida krusei* (recently reclassified as *Pichia kudriavzevii*) displays innate resistance to fluconazole, and certain isolates demonstrate diminished susceptibility to amphotericin B (Jamiu et al., 2021; Nguyen et al., 2024). This pathogen is frequently encountered among patients with hematologic malignancies or those undergoing hematopoietic stem-cell transplantation, with reported mortality rates as high as 67%. The emergence of breakthrough *C. krusei* infections during azole prophylaxis underscores the urgent need for alternative therapeutic strategies (Nguyen et al., 2024).

#### 2.1.2 Mould

*Aspergillus* species represents the most common mould infection genus, including *Aspergillus fumigatus*, *Aspergillus flavus*, *Aspergillus niger*, and *Aspergillus terreus*. Invasive aspergillosis (IA) predominantly affects the lungs but may disseminate to the central nervous system, gastrointestinal tract, and other anatomical sites. In general ICU populations, the incidence of invasive aspergillosis ranges from 1% to 5% (Chen et al., 2022). The average annual mortality attributable to IA is approximately 85.2%, though outcomes vary substantially by underlying disease status, infecting species, and geographic region. Chronic pulmonary aspergillosis is associated with a mortality rate of roughly 18.5%, whereas overall mortality among patients with COPD complicated by IA ranges from 43% to 72%, with attributable mortality reported between 83% and 90% (Bretagne et al., 2022). A recent meta-analysis focusing on critically ill patients with influenza demonstrated that influenza-associated pulmonary aspergillosis (IAPA) is strongly linked to markedly increased ICU mortality, with case-fatality rates frequently exceeding 50% across cohorts from Europe, North America, and Asia (Lu et al., 2024).

Mucormycosis represents a heterogeneous disease, with *Rhizopus* spp., *Mucor* spp., and *Lichtheimia* spp. constituting the most common causative organisms (Skiada et al., 2020). Susceptible populations include diabetic patients, solid organ transplant recipients, patients with haematological malignancies, and immunocompetent individuals with various cutaneous and soft tissue injuries. Global prevalence of mucormycosis ranges from 0.005 to 1.7 cases per million population, with India reporting the highest incidence globally (0.14‰) (Prakash and Chakrabarti, 2019). Delayed diagnosis and treatment of mucormycosis contribute to its substantial mortality rate, with 90-day mortality reaching 52% (Patel et al., 2020).

*Fusarium* species are ubiquitous in air and soil, causing invasive fusariosis through respiratory routes or damaged skin (Al-Hatmi et al., 2016). The most prevalent fungal species responsible for human infections are the *Fusarium solani* species complex (FSSC), accounting for approximately 50% of cases, and the *Fusarium oxysporum* species complex (FOSC), accounting for approximately 20% (Nucci and Anaissie, 2023). Immunocompetent individuals may develop superficial infections such as onychomycosis and keratitis, whereas immunocompromised hosts frequently develop invasive disease. Park et al. conducted a retrospective analysis at a tertiary medical center in Seoul, Korea, from 2005 to 2020, revealing that among patients with invasive fusariosis, 81% had a history of immunosuppressant use and 62% had haematological malignancies. Disseminated fusariosis (54%) and invasive pulmonary disease (23%) represented the most common clinical manifestations. The 28-day and in-hospital mortality rates for invasive fusariosis were 40% and 52%, respectively (Park et al., 2023).

Overall, while non*-Aspergillus* infections demonstrate relatively low incidence, they are associated with high mortality rates. Consequently, continual environmental control and heightened awareness of relevant fungal infections in immunocompromised patients are imperative, with early definitive diagnosis and targeted therapy being crucial for patient prognosis.

### 2.2 Increasing frequency of antifungal resistance

Antifungal agents comprise four principal chemical categories: azoles, echinocandins, polyenes, and flucytosine. Azole antifungals inhibit cytochrome P-450-mediated ergosterol biosynthesis, resulting in fungal cell membrane disruption. Echinocandins inhibit cell wall biosynthesis by blocking (1→3)-β-D-glucan synthase (GS). Amphotericin B, primarily administered as a liposomal formulation (L-AmB), represents the only systemically applicable polyene, exerting fungicidal effects through interaction with ergosterol, membrane extracellular aggregation, and/or pore formation (e37). Flucytosine undergoes deamination in fungal cells and inhibits fungal nucleic acid biosynthesis.

Recent years have witnessed alterations in non-albicans *Candida* susceptibility to antifungal agents. Global antifungal surveillance analysis indicates that between 2016 and 2017, over 99% of *C. albicans* isolates remained susceptible to fluconazole and voriconazole. *C. glabrata* demonstrated overall fluconazole resistance of 6.5%, highest in the United States (13.0%), while voriconazole resistance increased to nearly 50% (Castanheira et al., 2020). *C. tropicalis* resistance to fluconazole and voriconazole has continuously increased from below 10% in 2009 to exceeding 30% (Xiao et al., 2018; Fan et al., 2019; Castanheira et al., 2020; Borjian Boroujeni et al., 2021).

*C. auris* exhibits extensive multidrug resistance, demonstrating resistance to nearly all first-line antifungal agents. In 2019, the CDC classified *C. auris* among five pathogens posing urgent threats to public health and as the first fungal pathogen in the urgent antimicrobial resistance threat category (CDC, 2022). *C. auris* demonstrates fluconazole resistance exceeding 90%, amphotericin B resistance of approximately 30%, and relatively lower echinocandin resistance at approximately 7% (Lockhart et al., 2017). Research on *C. auris* resistance mechanisms reveals an outer cell wall layer with high mannopolysaccharide density and low structural complexity, contributing to reduced innate immune recognition. Additionally, *C. auris* exhibits weaker activation of MAPK signalling pathways controlling pro-inflammatory cytokine and chemokine expression in macrophages, facilitating evasion of host immune responses (Wang et al., 2022). Azole-resistant *C. auris* strains demonstrate ERG11(K143R) and CDR1(V704L) gene mutations. Echinocandin-resistant strains initially exhibit FKS1(S639Y) mutations, followed by distinctive FKS1(F635C) mutations. Flucytosine-resistant strains manifest FCY1, FUR1, and ADE17 mutations. Pan-resistant *C. auris* isolates demonstrate uridine phosphoribosyltransferase deletion (FUR1[1Δ33]) and FUR1 expression elimination (Lockhart et al., 2017; Jacobs et al., 2022). Amphotericin B resistance mechanisms require further investigation. For *C. auris* infections, echinocandins may represent the optimal initial therapeutic approach. The multidrug resistance profile and high mortality associated with *C. auris* necessitate heightened vigilance, with accurate species identification being crucial for appropriate treatment and prevention.

## 3 Early detection techniques for fungal infection

**Figure 1:**
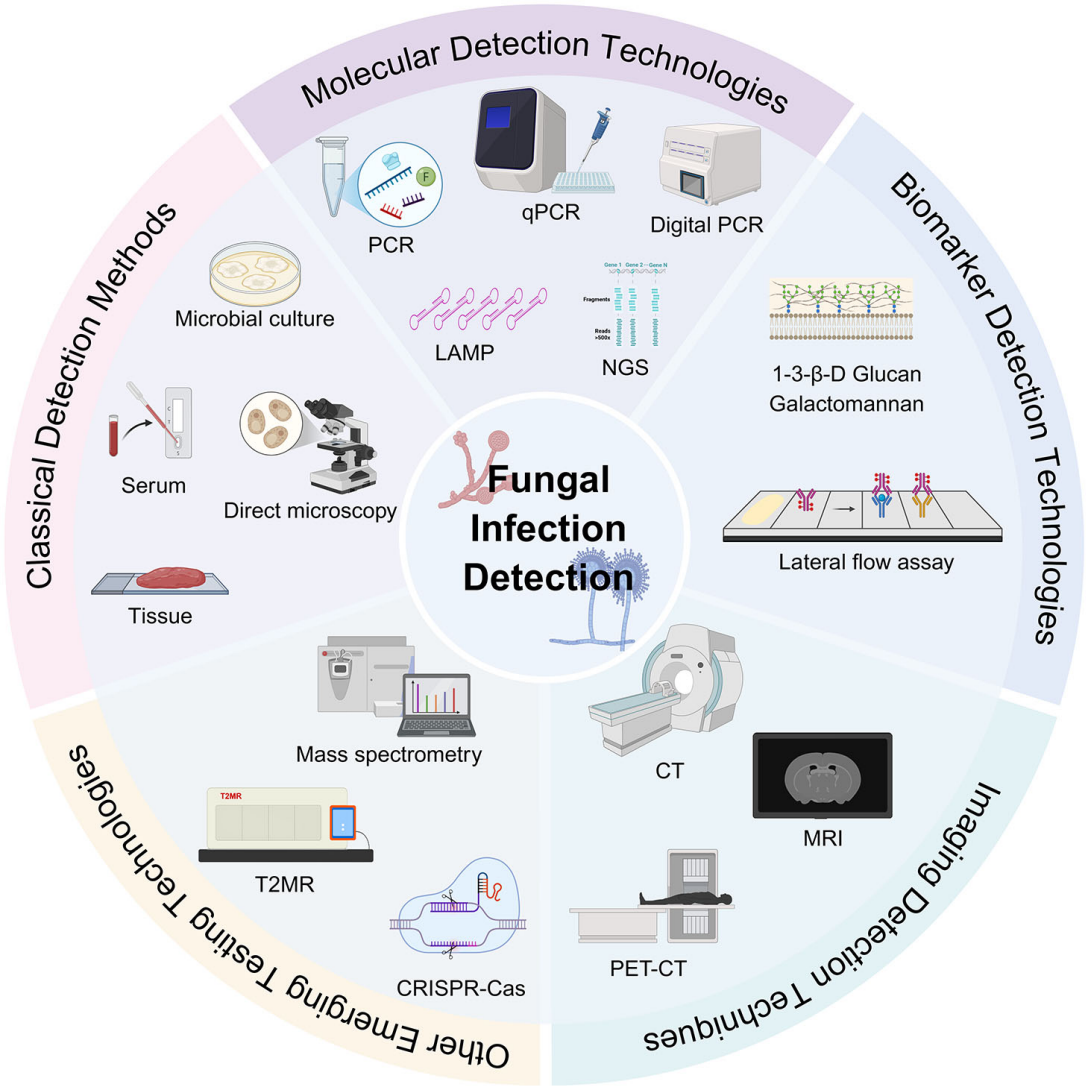
Early detection techniques for fungal infection. PCR, Polymerase chain reaction; qPCR, Real-time fluorescence quantitative PCR; LAMP, Loop-mediated isothermal amplification; NGS, Next-generation sequencing; PET-CT, positron emission tomography/Computed tomography; MRI, magnetic resonance imaging; CRISPR, Clustered regularly interspaced short palindromic repeats.

The diagnosis of IFI is defined according to the criteria of the European Organisation for Research and Treatment of Cancer/Invasive Fungal Infections Cooperative Group and the National Institute of Allergy and Infectious Diseases Fungal Disease Study Group (EORTC/MSG) (Donnelly et al., 2019). The gold standard for the diagnosis of IFI remains the microbiological culture method, and other classical tests include histopathological examination as well as direct microscopic examination; however, traditional tests have the drawbacks of long testing period, subjectivity, and low accuracy. Serological examination, as an important supplement to the traditional detection methods, can improve the detection efficiency, but it is susceptible to false positives due to the interference of various factors, and cannot achieve accurate strain identification. With the advancement of molecular technology, new molecular diagnostic platforms are emerging, and rapid molecular diagnostic methods can improve the ability of rapid intervention, reduce the occurrence of fatal events, and bring new opportunities for the early diagnosis of clinical fungal infections (**Figure 1**).

### 3.1 Molecular detection technologies

Molecular detection technologies encompass traditional polymerase chain reaction (PCR), real-time fluorescent quantitative PCR (qPCR), loop-mediated isothermal amplification (LAMP), and high-throughput sequencing techniques (Pham et al., 2024). These methodologies, predicated on the detection of fungal DNA or RNA in clinical specimens, are critical for selecting appropriate antifungal therapy.

#### 3.1.1 Polymerase chain reaction technologies

PCR has been extensively employed in IFI diagnostics, with numerous PCR detection methods rapidly emerging in recent years. PCR represents one of the most commonly utilised molecular detection methodologies, detecting fungal infections through amplification of specific DNA sequences. The EORTC/MSGERC guidelines have incorporated broad-spectrum PCR as a component of IFD diagnostic criteria. PCR methodologies are applicable to various microorganisms, including mould, dermatophytes, and yeasts. Clinical adoption rates are highest for *Aspergillus* PCR, consequently incorporated into the EORTC/MSGERC guidelines (Donnelly et al., 2019).

Conventional PCR relies on end-point detection of amplified fungal DNA via gel electrophoresis, which limits its ability to distinguish viable from non-viable pathogens and precludes quantification of fungal burden. In contrast, quantitative real-time PCR (qPCR) employs fluorescence-based detection to continuously monitor the amplification process, enabling both precise measurement of fungal load and dynamic assessment of therapeutic response. qPCR provides information regarding fungal burden, crucial for monitoring responses to antifungal therapy. qPCR detection of *Mucorales* demonstrates sensitivity between 80% and 90% (Rocchi et al., 2024). PCR detection has proven relatively complex in certain field conditions and specialised environments due to expensive thermal cycling, requirements for trained personnel, and gel electrophoresis equipment.

#### 3.1.2 Isothermal amplification technologies

LAMP represents a more recent amplification technology, performing DNA amplification at constant temperature, characterised by operational simplicity and absence of complex instrumentation requirements (Notomi et al., 2015). The LAMP assay can produce results within 30–60 minutes. It has high sensitivity for *Candida*, *Aspergillus* and *Mucor*, and is particularly suitable for bedside testing due to its low equipment requirements. Compared to PCR, LAMP offers multiple advantages, including high sensitivity, specificity, and rapid turnaround time. Research indicates that LAMP technology demonstrates superior sensitivity and specificity for *Candida* detection compared to traditional PCR methodologies. However, LAMP detection necessitates complex and cumbersome primer design; future optimisation of primer design is required to achieve more precise, rapid, and sensitive detection objectives.

Closed dumbbell-mediated isothermal amplification (CDA) represents a novel nucleic acid isothermal amplification technology, characterised by utilisation of Closed Dumbbell Probes to achieve highly specific, sensitive target nucleic acid amplification. CDA detection technology employs conserved fungal sequences as targets for specific identification and amplification, such as the ITS region in *Candida* species (Zhang et al., 2025).

#### 3.1.3 Sequencing technologies

High-throughput sequencing (HTS) represents a significant breakthrough in molecular detection. HTS technologies facilitate broad, culture-independent pathogen detection and have demonstrated clinical utility in identifying mixed or uncommon fungal infections that may evade targeted assays. Compared to traditional sequencing technologies, HTS enables comprehensive genomic analysis of numerous samples within a short timeframe, characterised by high throughput, sensitivity, and accuracy. The increasing prevalence of next-generation sequencing (NGS) or whole-genome sequencing (WGS) technologies significantly enhances our understanding of fungal phylogeny, epidemiology, pathogenesis, mycobiome/microbiome, and host interactions, while providing novel approaches for antifungal resistance diagnosis and treatment (White et al., 2022). NGS, including metagenomic sequencing directly from blood or bronchoalveolar lavage, provides unbiased detection even when prior antifungal exposure suppresses culture growth. NGS can also be used to characterise antifungal resistance markers, although turnaround time, cost and bioinformatics requirements currently limit widespread routine use. WGS further supports outbreak investigation and epidemiological surveillance by enabling strain-level resolution, transmission mapping and identification of resistance-associated mutations such as those linked to azole resistance in *Aspergillus fumigatus* and *Candida auris*. Carlos et al. performed fungal sequencing in patients diagnosed with IFI, demonstrating sensitivity and specificity of 96.6% (95% CI, 87.4%–99.4%) and 98.2% (95% CI, 89.4%–99.9%), respectively, in confirmed patients (Gomez et al., 2017).

In summary, advancements in molecular detection technologies provide novel approaches for early diagnosis and treatment of infectious diseases. qPCR and LAMP demonstrate greater speed, sensitivity, and specificity compared to traditional PCR technologies, while nucleic acid sequencing provides information regarding fungal species, crucial for developing personalised treatment strategies. However, these detection technologies have limitations, including specialised equipment requirements, potential false-positive results due to contamination, and inability to distinguish between viable and non-viable fungal cells. Additionally, significant barriers persist in implementing NGS and mNGS technologies in diagnostic laboratories. With continued technological advancements, molecular detection will play an increasingly important role in early detection of fungal infections.

### 3.2 Biomarker detection technologies

Fungal-specific antigen detection represents one of the commonly employed technologies for clinical auxiliary diagnosis of IFI, encompassing cell wall polysaccharide component determination, metabolite determination, and biomarker detection methodology platforms. Traditional detection methods include assays for 1,3-β-D-glucan (BDG) and galactomannan (GM). BDG represents a polysaccharide component of most fungal cell walls, including *Candida* species, *Pneumocystis jirovecii*, *Aspergillus* species, and *Fusarium* species. Currently, an FDA-approved detection method exists—the Fungitell assay (Associates of Cape Code, Inc., East Falmouth, MA, USA). BDG testing has been extensively employed for IC diagnosis and therapeutic guidance. In IC diagnosis, sensitivity ranges between 70% and 90%, but specificity demonstrates poorer performance at 50% to 70% (Haydour et al., 2019). Notably, *Mucorales* cell walls lack glucans, while *Cryptococcus* cell walls, although containing glucans, primarily comprise α-glucans and β-1,6-glucans. Consequently, BDG detection is unsuitable for *Cryptococcus* and *Mucorales* detection.

GM represents another cell wall biomarker for IFI, produced by various fungi including *Aspergillus*, *Penicillium*, *Paecilomyces*, *Lichtheimia*, and *Histoplasma* species. The Platelia *Aspergillus* enzyme immunoassay (Bio-Rad, Marnla cooche, France) represents an FDA-approved method for *Aspergillus* GM detection, demonstrating sensitivity and specificity of 78% and 93%, respectively, in bronchoalveolar lavage fluid (BALF) (de Heer et al., 2019). Traditional ELISA detection is limited by prolonged processing time and stringent detection environment requirements, consequently not practically implemented in most hospitals.

Lateral flow chromatography testing technology represents an emerging point-of-care detection method for invasive aspergillosis, with two currently developed assays: the AspLFD lateral flow device (LFD) by OLM Diagnostics (Newcastle upon Tyne, UK) and the sōna *Aspergillus* galactomannan lateral flow assay (LFA) by IMMY (Norman, OK, USA) (Mercier et al., 2020). These typically target *Aspergillus* cell wall GM components, enabling serum antigen detection within 15 minutes to 1 hour. They demonstrate excellent performance in both serum samples and BALF, with BALF sensitivity of 0.73-0.94 and specificity of 0.8-0.9 (Mercier et al., 2020; Fan et al., 2025).

### 3.3 Imaging technologies

Computed tomography (CT), owing to its high resolution and rapid imaging capabilities, has become an essential tool for evaluating pulmonary infections. The most frequently employed pulmonary imaging modality in clinical practice is high-resolution computed tomography (HRCT). Common manifestations of IPA on HRCT include the “halo sign”, “air crescent sign”, and “cavitation” (Barac et al., 2023). Early radiological manifestations of IPA typically include ground-glass opacities and small nodules, which may progress to larger nodules with the halo sign, consolidations, cavitations accompanied by the air-crescent sign, and ultimately residual fibrotic remodeling (Alexander et al., 2021). During the COVID-19 pandemic, the incidence of invasive fungal infections in critically ill patients increased significantly, particularly COVID-19-associated pulmonary aspergillosis (CAPA) and COVID-19-associated pulmonary mucormycosis (CAPM) (Koulenti et al., 2023). Pulmonary mucormycosis predominantly occurs in patients with diabetic ketoacidosis, solid-organ transplantation, or profound immunosuppression. CT typically reveals large areas of parenchymal consolidation (often >4 cm), cavitation, and the reverse halo sign (RHS)—a central ground-glass opacity encircled by a rim of consolidation—which is observed more frequently in pulmonary mucormycosis than in IPA (Jang et al., 2025). CT provides sensitive detection of pulmonary nodules, infiltrates, and other structural abnormalities, facilitating early recognition of radiological features suggestive of invasive fungal infection.

Magnetic resonance imaging (MRI), as another crucial imaging technology, offers unique advantages in detecting central nervous system, sinus, deep soft tissue, and other deep-seated fungal infections. MRI provides more detailed soft tissue imaging, facilitating identification of infectious foci and their impact on surrounding tissues, which is essential for comprehensive assessment of critically ill patients. Research indicates that MRI effectively enhances early diagnosis of acute invasive fungal rhinosinusitis (Voruz et al., 2025).

Positron emission tomography-computed tomography (PET-CT) combines the advantages of metabolic functional imaging (PET) with anatomical structural imaging (CT), capable of revealing metabolically active infectious or inflammatory lesions throughout the body by tracking radioactively labeled glucose analogs (such as ^18^F-FDG) (Kung et al., 2019). In invasive fungal infections, PET-CT holds significant value for detecting disseminated systemic infections (such as candidemia, disseminated invasive aspergillosis) or occult lesions. For example, in immunocompromised patients, PET-CT sensitively identifies deep tissue infections (such as pancreatic candidiasis, *Aspergillus* myocarditis, arthritis) that are difficult to detect with conventional imaging, while assessing infection extent and activity (Xue et al., 2024; Kim et al., 2025; Ravindra et al., 2025). Additionally, PET-CT can be integrated with microbiological or molecular detection results to optimise antifungal treatment duration and intensity. However, clinical application of PET-CT is limited by high costs, radiation exposure, and non-specific uptake (tumours or non-infectious inflammation may interfere with result interpretation).

Overall, innovative imaging technologies provide crucial support for early diagnosis in critically ill patients, assisting clinicians in making more accurate diagnoses at early stages, thereby facilitating timely implementation of effective therapeutic measures to reduce patient mortality and complication rates. With continued development and application of imaging technologies, additional innovative approaches are anticipated to be introduced into clinical practice, providing more comprehensive support for management of critically ill patients.

### 3.4 Emerging detection technologies

#### 3.4.1 T2 magnetic resonance imaging

T2 Magnetic Resonance (T2MR) represents a miniaturised magnet-based diagnostic methodology capable of detecting various targets, including molecular targets (such as DNA), immunodiagnostics (such as proteins), and a broad spectrum of haemostasis markers. T2MR is the first technology capable of rapidly and accurately detecting molecular targets in samples without time-consuming and labour-intensive purification or extraction of target molecules from samples. T2 Biosystems, operating on the fully automated T2Dx instrument, represents an FDA-approved rapid diagnostic method enabling sensitive and specific detection of *Candida* pathogens directly from whole blood without culture or nucleic acid extraction steps. All detection steps, from sample to result, are performed automatically on the T2Dx instrument. Compared to PCR, T2MR demonstrates significant advantages in speed and limit of detection (LOD). T2MR can detect microorganisms at densities as low as 1 CFU/ml, whereas traditional PCR methods typically require 100-1,000 CFU/ml. T2 *Candida* employs T2MR technology for qualitative detection of five *Candida* species by category: *Candida albicans* and/or *Candida tropicalis* (A/T), *Candida parapsilosis* (P), *Candida krusei* and/or *Candida glabrata* (K/G) (Neely et al., 2013; Mylonakis et al., 2015). Camp et al. conducted a prospective, observational multicentre study analysing blood samples from patients with suspected candidemia from August 2018 to April 2020. The study demonstrated that conventional blood culture exhibited a sensitivity of 40.5% (95% CI: 23–58%), whereas the T2 assay achieved a substantially higher sensitivity of 73.0% (95% CI: 56–86%) (Camp et al., 2024). These findings indicate that T2 technology may enable earlier detection of candidemia, particularly in cases missed by conventional culture.

#### 3.4.2 Mass spectrometry-based detection technologies

In the field of fungal infections, mass spectrometry-based detection technologies include matrix-assisted laser desorption ionisation time-of-flight mass spectrometry (MALDI-TOF MS), mass spectrometry detection systems (MS-DS), and mass spectrometric analysis of fungal metabolic characteristics. Through laser irradiation of co-crystalline films formed by colonies and matrices, matrices absorb energy from lasers and ionise proteins within colonies. The signal intensity of these ionised proteins reaching detectors provides mass spectra, which are compared with reference database spectra for microbial identification (Chen et al., 2021). MALDI-TOF MS provides a faster, more accurate method for identifying fungal species isolated from patients, shortening the microbial identification process by approximately one day, becoming indispensable in clinical mycology laboratories. Currently, four commercial systems are available: MALDI Biotyper (Bruker Daltonics, Bremen, Germany), AXIMA-SARAMIS (AnagnosTec, Potsdam-Golm, Germany), Andromas SA (Paris, France), and VITEK MS (bioMérieux, Marcy l’Etoile, France) (Lohmann et al., 2013; Tsuchida et al., 2020). MS-DS demonstrates sensitivity and specificity of 51% and 87%, respectively, for IC detection. This detection method can also differentiate sera from IA patients and neutropenic control groups (P ≤ 0.0009), with sensitivity and specificity of 64% and 95%, respectively, for IA detection (Cornu et al., 2019).

#### 3.4.3 Clustered regularly interspaced short palindromic repeats

CRISPR/CRISPR-associated proteins represent a form of adaptive immune defence present in most bacteria and archaea, protecting them from phage infection, viruses, and other foreign genetic elements (65). CRISPR-based technologies establish foundations for novel fungal diagnostic tools capable of rapidly and accurately detecting genetic material without specialised expertise or equipment. CRISPR-Cas9 demonstrates high specificity, and this toolkit has been applied to detect *Candida* species, *Cryptococcus neoformans*, *Aspergillus fumigatus*, and *Mucorales* (Matsuo et al., 2024). The application of CRISPR technology holds significant value for research on fungal pathogenesis and antimicrobial resistance. With further development of gene drives, base editing, and non-editing applications, this technology is anticipated to play more substantial roles in basic research (such as host-pathogen interactions) and clinical applications (such as rapid diagnostics, antimicrobial resistance monitoring), providing new tools for addressing global challenges of fungal infections.

In summary, current fungal infection detection technology systems encompass molecular detection, biomarker detection, imaging, and other emerging detection technologies. These technological advancements provide multidimensional support for early diagnosis of IFI in critically ill patients, potentially significantly improving diagnostic accuracy and timeliness, thereby enhancing patient prognosis. However, these technologies exhibit various deficiencies, including requirements for specialised equipment and personnel, false-positive risks, inability to distinguish between viable and non-viable fungi, and relatively high costs. Future focus should be directed toward technological simplification, sensitivity enhancement, detection time reduction, and integrated application of multiple technologies to provide more comprehensive support for early diagnosis of fungal infections in critically ill patients, thereby improving patient prognosis.

## 4 Artificial intelligence technologies for a paradigm shift in diagnosis and treatment

**Figure 2:**
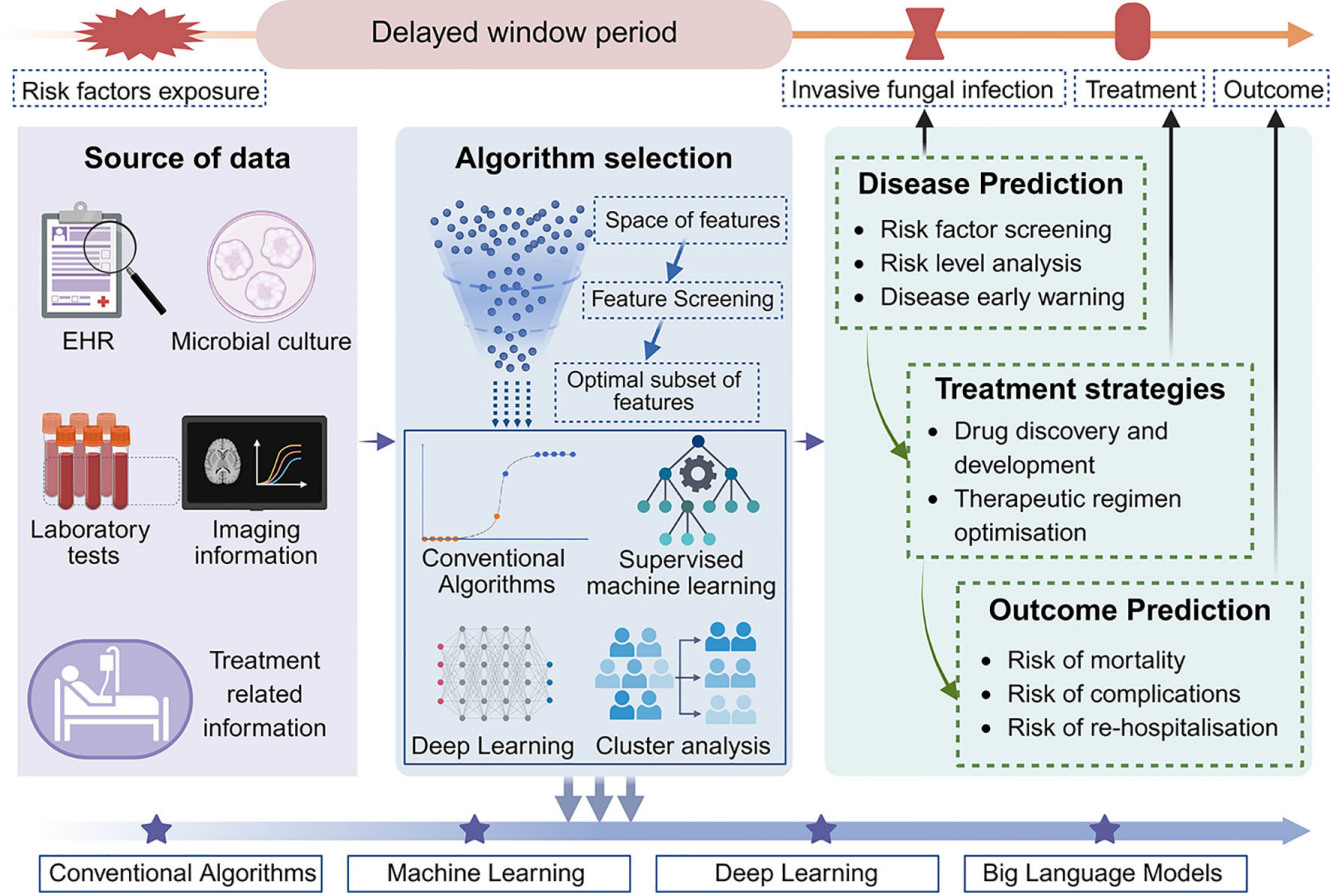
Application of artificial intelligence techniques in the field of invasive fungal infections. EHR, Electronic Health Record.

The safety and early diagnosis of IFI is a central challenge in routine clinical practice and a key foundation for targeted therapies. There is a delayed window of time between exposure to risk factors and the onset of infection in IFI, which offers the possibility of early risk assessment of patients for the development of invasive fungal infections, and early prophylaxis and empirical antifungal treatment of high-risk patients after risk assessment, potentially to the benefit of the patient. In the 21st century, machine learning (ML) algorithms and deep learning (DL) algorithms have flourished, and the advantages of these algorithms, such as their superior information retrieval capability, storage capacity, and accuracy, have laid a good foundation for their application and development in the healthcare field, which has brought about revolutionary changes in the healthcare model (Deo, 2015; Bzdok et al., 2018; Handelman et al., 2018). Predictive models based on AI algorithms can be used for early diagnosis of diseases and improve diagnostic accuracy; treatment decision models based on deep learning create the possibility of personalised medicine. We present a summary of research related to AI techniques in the field of invasive fungal infections, which can be categorised into three directions, namely prediction of the risk of disease occurrence (Poissy et al., 2020; Bhavani et al., 2020; Li et al., 2018; Playford et al., 2016; Guillamet et al., 2015; Stanzani et al., 2019; Jin et al., 2025; Yuan et al., 2021; Mayer et al., 2022; Yan et al., 2022; Filigheddu et al., 2024; Meng et al., 2024; Su et al., 2024; Cao et al., 2024; Wang et al., 2023; Ripoli et al., 2020), prediction of the effectiveness of therapeutic decision-making (Ostrosky-Zeichner et al., 2017; Zhao et al., 2021; Rauseo et al., 2022), and prediction of outcome (Li et al., 2022; Vaquero-Herrero et al., 2017; Bassetti et al., 2017; Du et al., 2024; Gao et al., 2022) (**Table 1** and **Figure 2**).

**Table 1:** Research on artificial intelligence technology in the field of invasive fungal infections.

Research field	Algorithm type	Performance of the best model	Model contents	Sample size	Validation	References
Risk prediction	Logistic regression; Stepwise regression	0.77^a^	Risk prediction model for candidaemia in ICU and outside patients; Candidaemia outcome prediction model.	603	Internal	(Poissy et al., 2020)
	Logistic regression; Gradient Boosting Machine	0.88^a^	Risk prediction model for bacteremia and fungalemia in hospitalized patients.	76688	Internal	(Bhavani et al., 2020)
	Logistic regression	0.73^a^	Risk prediction model for invasive fungal infection in ICU patients.	141	Internal	(Li et al., 2018)
	Logistic regression	0.82^a^	ICU patients were divided into high risk, medium risk and low risk groups according to the IC risk score.	6685	Internal	(Playford et al., 2016)
	Logistic regression	0.80^a^	Development of a predictive model for candidemia in hospitalized patients with severe sepsis and septic shock.	2597	Internal	(Guillamet et al., 2015)
	Logistic regression	0.85^a^	A risk prediction model for invasive mycosis (IMD) within 60 days of admission to hospital for haematological malignancies.	2187	Internal	(Stanzani et al., 2019)
	LASSO regression; Logistic regression; χ2 automatic interaction detection (CHAID); Decision tree	0.8^b^	To assess the risk of IFI in patients admitted to the ICU with sepsis.	549	–	(Jin et al., 2025)
	XGBoost; SVM; Random forest; ExtraTrees (ET); Logistic regression	0.92^a^	Predicting candidaemia in patients with new-onset systemic inflammatory response syndrome.	8002	Internal	(Yuan et al., 2021)
	Random forest	Not reported	The risk factors of invasive candidiasis (IC) and non-IC, intraspecies IC and non-IC, and interspecies IC were analyzed in the positive cases of *Candida* laboratory.	116725	Internal	(Mayer et al., 2022)
	Logistic regression	0.844^a^	To investigate the value of a machine learning model constructed on the basis of CT imaging histological features combined with clinical factors in identifying IFI from bacterial pneumonia in patients with haematological malignancies.	235	Internal	(Yan et al., 2022)
	Staged tree models	Not reported	Impact of chest CT findings and radiological patterns on patient prognosis in patients with *Aspergillus* and other filamentous fungal IFI (AFF-IFI).	146	–	(Filigheddu et al., 2024)
	XGBoost; Logistic regression; Support vector machine; Recurrent neural network; Random forest	0.87^a^	Machine Learning Predictive Models for Early Candidaemia Diagnosis in ICU Patients.	334	Internal External	(Meng et al., 2024)
	Logistic regression	0.81^a^	Early identification of secondary IFI cases in COVID-19 patients in the ICU.	262	Internal	(Su et al., 2024)
	Random forest; Logistic Regression; K-Nearest Neighbor; Decision Tree; eXtreme Gradient Boosting; Single-hidden-layer neural network	0.88^a^	An early prediction model for the development of invasive fungal infections in patients based on ICU patients in the MIMIC database.	26346	Internal	(Cao et al., 2024)
	Random forest	0.874^b^	Predicting the risk of early candidaemia in internal medicine wards based on a random forest model.	295	–	(Ripoli et al., 2020)
	Deep learning	0.95^a^	Deep learning-based early diagnostic model for invasive non-*Aspergillus* using early clinical features of patients and chest CT-related information.	–	Internal	(Wang et al., 2023)
Therapeutic decision-making	LASSO regression	0.65^b^	Risk assessment model for predicting failure in patients with candidaemia treated with fluconazole.	987	Internal	(Ostrosky-Zeichner et al., 2017)
	Logistic regression	0.91^a^	Prediction of adverse events and fluconazole concentration prediction model in renal transplant patients receiving fluconazole therapy.	93	Internal	(Zhao et al., 2021)
	Logistic regression; Stepwise regression	0.79^b^	A predictive model for fluconazole resistance in patients with candidaemia.	539	Internal	(Rauseo et al., 2022)
	Deep learning	91.5%^c^	Accelerate the identification of antifungal peptides.	–	Internal	(Yin et al., 2025)
	Deep learning algorithm Transfer learning	0.96%^c^	Identification of Natural Antifungal Peptides.	–	Internal External	(Sharma et al., 2022)
Outcome Prediction	Logistic regression; Random forest; Support vector machine	0.92^a^	Prognostic model for patients with invasive *Candida* infections and bacterial bloodstream infections.	246	Internal	(Li et al., 2022)
	Logistic regression	Not reported	Risk prediction model for outcome of candidaemia.	189	Internal	(Vaquero-Herrero et al., 2017)
	Logistic regression; Stepwise regression	0.75^a^	Clinical characteristics and outcome prediction models in cirrhotic patients with combined candidaemia and intra-abdominal *Candida* infection.	241	Internal	(Bassetti et al., 2017)
	COX regression	0.853^b^	A prognostic model for patients with non-neutropenic invasive pulmonary aspergillosis.	210	Internal	(Du et al., 2024)
	Logistic regression; Random forest; Support vector machine	0.85^a^	Prognostic model for candidemia and bacteremia.	367	Internal	(Gao et al., 2022)

ICU, intensive care unit. a: Area Under the Subject Operating Curve (AUROC). b: C-statistic. c: Precision.

### 4.1 Traditional methods and prediction rule

In the clinical domain, prediction tool development has traditionally been based on background knowledge and statistical models, using parametric (such as logistic regression) or semi-parametric (such as Cox regression) approaches. Poissy et al. conducted a prospective multicentre matched case-control study incorporating 192 candidemia patients (Poissy et al., 2020). Using logistic regression and stepwise regression methods to screen for IC risk factors, the study identified total parenteral nutrition, acute kidney injury, cardiac disease, previous septic shock, and aminoglycoside exposure as independent risk factors for candidemia in ICU populations; they subsequently developed a candidemia prediction score for ICU patients with an AUC of 0.768. Playford et al. performed a multicentre prospective cohort study of 6,685 ICU patients in Australia, constructing an invasive candidiasis risk prediction model that stratified ICU patients into high-risk, moderate-risk, and low-risk groups, facilitating more targeted early antifungal therapy implementation (Playford et al., 2016). This model demonstrated an AUC of 0.82. Guillamet et al. utilised information from 2,597 patients to establish a clinical prediction rule for candidemia in patients with severe sepsis and septic shock based on logistic regression methodology. This prediction rule achieved an AUC of 0.798 (Guillamet et al., 2015). Stanzani et al. employed clinical information from 1,944 adult patients with haematological malignancies to construct a model using logistic regression methodology to predict patients’ risk of developing invasive mould disease (IMD) within 60 days after hospital admission for haematological malignancy treatment. This model demonstrated an AUC of 0.85 (Stanzani et al., 2019). Rauseo et al. developed a prediction model based on 539 candidemia patients using multivariate logistic regression and stepwise regression methodologies to identify patient populations with lower fluconazole resistance risk. This model demonstrated a c-statistic of 0.771 and sensitivity of 90.3% (Rauseo et al., 2022). Ostrosky-Zeichner et al. developed a risk assessment model predicting fluconazole treatment failure in candidemia patients (Ostrosky-Zeichner et al., 2017). This predictive model identified high-risk patients for fluconazole failure, demonstrating the potential value and feasibility of personalised candidemia treatment, with a model c-statistic of 0.65. Zhao et al. prospectively analysed 93 renal transplant patients receiving voriconazole treatment, employing logistic regression to identify predictors of adverse events while determining that CYP2C19 phenotype, platelet count, haemoglobin, and concomitant esomeprazole use were determinants of voriconazole trough concentrations (Zhao et al., 2021).

Collectively, these studies demonstrate that traditional statistical approaches—predominantly logistic regression–based models—have successfully yielded clinical prediction tools for invasive candidiasis and invasive mould infections across diverse high-risk populations. Such models consistently achieve moderate-to-good discriminative performance, underscoring their clinical utility in early risk stratification and timely initiation of antifungal therapy. Nonetheless, their dependence on a limited set of predefined clinical variables constrains their capacity to capture the biological complexity and heterogeneity inherent to invasive fungal infections, resulting in variable generalizability across healthcare settings and patient cohorts. Consequently, the integration of dynamic, high-dimensional clinical and laboratory parameters, and potentially multi-omics data, represents a highly promising avenue to advance individualized risk assessment.

### 4.2 Machine learning algorithms enhancing prediction model performance

Machine learning algorithms effectively model non-linear relationships between variables and outcomes, as well as non-linear relationships among predictive variables themselves. The establishment of disease early warning models using machine learning algorithms has been widely applied to early prediction of various diseases. Within the field of invasive fungal infections, Bhavani et al. employed logistic regression and gradient boosting machine (GBM) models to develop prediction algorithms for bacteremia and fungemia among hospitalized patients. Prior history of fungal disease and the interval between admission and the first positive blood culture emerged as the most influential predictors of fungemia. The GBM model demonstrated robust performance, achieving an AUC of 0.88 in internal validation, outperforming conventional clinical scoring systems such as the SIRS criteria, the Infection Probability Score, and the Modified Early Warning Score, and exhibiting superior discriminatory capacity (Bhavani et al., 2020). Meng et al. constructed a machine-learning–driven prediction model for candidemia in intensive care unit patients and conducted external validation involving 77 individuals. The random forest model yielded the strongest performance, maintaining an AUC of 0.87 in the external cohort, indicative of promising generalizability (Meng et al., 2024). Wang et al. developed an early diagnostic model for invasive pulmonary aspergillosis (IPA-NET) based on deep learning, utilising patient information including CT images. IPA-NET provides a non-invasive, objective, and reliable methodology for early IPA diagnosis, demonstrating accuracy of 89.7%, sensitivity of 0.88, specificity of 0.91, and AUC of 0.95 (Wang et al., 2023). Ripoli et al. utilised data from 295 patients to develop a random forest-based early prediction model for candidemia in medical wards, achieving an AUC of 0.847 (Ripoli et al., 2020).

### 4.3 Artificial intelligence methods supporting treatment decisions

Beyond early diagnosis, source control and early effective systemic antifungal therapy are crucial for successful management of IFD. Early appropriate antibiotic therapy effectively reduces patient mortality risk and economic burden while decreasing antimicrobial resistance development. Antifungal resistance represents an increasingly serious problem in fungal therapy. Some *Candida* species considered fully susceptible to fluconazole, such as *C. albicans*, *C. tropicalis*, and *C. parapsilosis*, may develop fluconazole resistance, while isolates of typically resistant non-albicans *Candida* species (such as *C. glabrata*) may maintain susceptibility.

Currently, clinical treatment decisions are primarily guided by species identification and antifungal susceptibility testing. However, microbiological culture cycles are relatively lengthy, unfavourable for early patient treatment. Utilising machine learning algorithms for patient risk discrimination, implementing prophylactic antifungal therapy for high-risk patients, and employing higher-level antifungal agents for potentially resistant cases may improve IFD patient survival rates. Yin et al. developed the Deep Learning-Quantitative Structure-Activity Relationship Empirical Screening (DL-QSARES) method, integrating deep learning and quantitative structure-activity relationship screening to design novel antifungal peptides *de novo*. This approach successfully identified 49 candidate antifungal peptides, whose anti-*Candida* activity was validated in both *in vitro* and *in vivo* experiments (Yin et al., 2025). Sharma et al. developed the Deep-AFPpred model using transfer learning and the 1DCNN-BiLSTM deep learning algorithm to identify antifungal peptides from protein sequences. Deep-AFPpred significantly outperformed other state-of-the-art AFP classifiers on both validation and test datasets, achieving accuracies of approximately 96% and 94% respectively (Sharma et al., 2022). Rozova et al. employed natural language processing techniques and various machine learning algorithms, integrating available electronic health record data including cytology and histopathology reports, to develop an automated IFI detection tool (Rozova et al., 2023).

### 4.4 Outcome prediction

Li et al. utilised data from 246 IC patients to construct prediction models using random forest, logistic regression, and support vector machine algorithms. The research identified primary death-associated risk factors including serum creatinine, serum albumin, CRP, PCT, total bilirubin levels, age, hospitalisation duration, ICU length of stay, leukocyte count, and neutrophil count (Li et al., 2022). The random forest-based prediction model demonstrated optimal performance with an AUC of 0.919. A multicentre retrospective cohort study investigated clinical characteristics and risk factors for adverse events in cirrhotic patients with concurrent candidemia and intra-abdominal *Candida* infections using logistic regression models and backward stepwise regression algorithms (Bassetti et al., 2017).

In our review, the vast majority of studies reported only internal validation, with very few conducting external validation in independent cohorts. Multiple factors likely contribute to this gap: diagnostic practices differ across regions and institutions, patient populations vary substantially, and the quality of data from disparate sources is often inconsistent. These objective limitations hinder the broader implementation of prediction models in clinical practice. Consequently, many investigations remain confined to the model development phase, and substantial progress is still required before these tools can be reliably deployed in real-world clinical settings. Additionally, many existing models in related fields use microbiological cultures or colonisation markers (e.g. airway *Aspergillus* isolation in ICU patients) as outcomes, which may reflect fungal colonisation rather than proven/probable IFI as per EORTC/MSGERC criteria. More precise differentiation between colonisation and invasive disease will likely require incorporating specific fungal biomarkers—such as serum or BAL galactomannan, β-D-glucan, and fungal PCR assays—into future algorithms, either as predictors or as part of composite outcome definitions.

In summary, research regarding invasive fungal infections remains in the exploratory phase, with relatively few studies employing machine learning for fungal infection prediction models, primarily utilising traditional algorithms. Additionally, current research in related fields may represent colonisation rather than invasive infection, necessitating further clarification of IFI definitions. Finally, retrospective studies may demonstrate significant discrepancies from clinical realities. Consequently, prospective research is required to further promote interdisciplinary application of artificial intelligence technologies in the IFI field, leveraging their advanced capabilities to drive intelligent development of clinical diagnosis and treatment for invasive fungal infections.

## 5 Conclusion

With deepening research into early diagnostic technologies for invasive fungal infections in critically ill patients, the medical community has achieved significant progress in identifying and managing these infections. Recent years have witnessed continuous emergence of novel biomarkers, imaging methodologies, and molecular detection techniques, providing expanded possibilities for early diagnosis. Application of these technologies has not only enhanced diagnostic accuracy and timeliness but also demonstrated immense potential for improving overall prognosis in critically ill patients. However, despite biomarkers demonstrating promising prospects in clinical applications, certain limitations persist regarding their specificity and sensitivity. Simultaneously, existing molecular detection technologies continue to face challenges in clinical popularisation, including cost, equipment requirements, and operational complexity.

In conclusion, with continued exploration of early diagnostic technologies for invasive fungal infections in critically ill patients, we anticipate these emerging technologies will bring additional breakthroughs to clinical practice, effectively improving prognosis in critically ill patients. Through strengthened interaction and application between different research areas, we will achieve greater accomplishments in this field, driving continuous medical advancement.

## Glossary

IFI: Invasive Fungal Infection
ICU: Intensive Care Unit
IC: Invasive Candidiasis
IA: Invasive Aspergillosis
IPA: Invasive Pulmonary Aspergillosis
COPD: Chronic Obstructive Pulmonary Disease
HIV: Human Immunodeficiency Virus
CDC: Centers for Disease Control and Prevention
FSSC: Fusarium solani Species Complex
FOSC: Fusarium oxysporum Species Complex
PCR: Polymerase Chain Reaction
qPCR: Quantitative Polymerase Chain Reaction
LAMP: Loop-Mediated Isothermal Amplification
CDA: Closed Dumbbell-mediated isothermal Amplification
HTS: High-Throughput Sequencing
NGS: Next-Generation Sequencing
WGS: Whole-Genome Sequencing
mNGS: Metagenomic Next-Generation Sequencing
ITS: Internal Transcribed Spacer
BDG: 1,3-β-D-Glucan
GM: Galactomannan
BALF: Bronchoalveolar Lavage Fluid
ELISA: Enzyme-Linked Immunosorbent Assay
LFD: Lateral Flow Device
LFA: Lateral Flow Assay
HRCT: High-Resolution Computed Tomography
CT: Computed Tomography
MRI: Magnetic Resonance Imaging
PET-CT: Positron Emission Tomography-Computed Tomography
CNS: Central Nervous System
T2MR: T2 Magnetic Resonance
LOD: Limit Of Detection
CFU: Colony-Forming Unit
MALDI-TOF MS: Matrix-Assisted Laser Desorption Ionization Time-Of-Flight Mass Spectrometry
MS-DS: Mass Spectrometry Detection System
CRISPR: Clustered Regularly Interspaced Short Palindromic Repeats
AUC: Area Under the Curve
SIRS: Systemic Inflammatory Response Syndrome
IPS: Institute for Healthcare Improvement Sepsis
MEWS: Modified Early Warning Score
CAPA: COVID-19-Associated Pulmonary Aspergillosis
CAPM: COVID-19-Associated Pulmonary Mucormycosis
IMD: Invasive Mould Disease
IFD: Invasive Fungal Disease
CRP: C-Reactive Protein
PCT: Procalcitonin
